# Deep learning approach for hybrid beamforming design in MU-MISO mmWave systems

**DOI:** 10.1038/s41598-026-35247-5

**Published:** 2026-02-04

**Authors:** Ebrahim Ghaith, Tamer Mekkawy, Ahmed A. Abouelfadl, Ashraf Mahran

**Affiliations:** 1 Avionics Department, Military Technical College, Cairo, 11511 Egypt; 2 Electrical Engineering Department, Military Technical College, Cairo, 11511 Egypt; 3 Electronics and Communications Department, Egypt University of Informatics, Cairo, 11511 Egypt

**Keywords:** Hybrid beamforming, MmWave, Deep learning, DeepMIMO, MU-MISO, Engineering, Mathematics and computing

## Abstract

Hybrid beamforming is a promising approach to alleviate hardware complexity in multi-user multiple-input single-output (MU-MISO) systems while maintaining high data rate performance. Unfortunately, hybrid beamforming architecture design is a challenging non-convex optimization problem due to stringent hardware constraints. However, traditional hybrid beamforming design methods, such as alternating minimization (AltMin) algorithms, rely on iterative optimization procedures that introduce heavy computational overhead and make them impractical for real-time applications. In this paper, we propose a deep learning (DL)-based hybrid beamforming method (DL-HBF) that aims to reduce computational latency while achieving acceptable sum-rate performance. Furthermore, we evaluate these methods based on a realistic channel model to ensure practical significance and their performance on imperfect channel state information (CSI). Additionally, we propose dataset generation procedures, which reduce the dataset creation and training overhead compared to existing DL-based hybrid beamforming methods that help in rapid deployment and scalability. Simulation results show that the proposed DL-HBF achieves an acceptable sum rate compared to traditional methods while reducing the computational complexity and maintaining robustness against channel estimation errors, which provides a practical solution for real-time hybrid beamforming for next-generation wireless systems.

## Introduction

Vehicular communications aim to improve safety and traffic efficiency, as well as real-time data collection and sharing, which demands high data rates and low latency. These demands motivate the use of millimeter waves (mmWave) for the next generations of wireless systems^1^. The mmWave communication technology represents a new approach to solve spectrum congestion problems because it possesses a wide frequency range between 30 and 300 GHz. Despite this potential, its signal suffers from high path loss and atmospheric absorption^2^. However, the short wavelength of mmWave makes it suitable for deploying a large number of antennas in a small dimension and opens the possibility of beamforming techniques to mitigate path loss and increase spectral efficiency^3^.

In practical mmWave systems, the base station (BS) is equipped with a large antenna array, while user terminals often employ a single antenna due to hardware complexity and power consumption. Therefore, it leads naturally to a multi-user multiple-input single-output (MU-MISO) architecture. Compared to single-user systems, MU-MISO mmWave systems have additional challenges related to inter-user interference management, simultaneously serving multiple single-antenna users via spatial beamforming, and beamforming design under stringent hardware constraints^4^. Consequently, the development of efficient beamforming strategies is essential for MU-MISO to achieve a high sum rate while maintaining low computational and hardware complexity.

The concept of analog beamforming was introduced in^5,6^, where analog phase shifters were used to set signal phases between individual antenna elements. However, this system architecture, which has a single RF chain, lacks the capability to support spatial multiplexing and is unsuitable for transmitting parallel data streams or simultaneously serving multiple users. Nonetheless, it has less hardware complexity and less power consumption. On the other hand, digital beamforming provides full control over magnitude and phase values within the baseband domain. Digital beamforming requires individual RF chains and both digital-to-analog (DACs) and analog-to-digital (ADCs) for every antenna element. Despite its flexibility and providing parallel data streams for multiple users, the use of separate RF chains, DACs, and ADCs for each antenna element leads to substantial hardware costs and high power consumption, making the approach unsuitable for large-scale antenna systems^7^. Therefore, a demand for hybrid beamforming has appeared, which represents an energy-efficient and cost-effective structure that integrates the advantages of analog and digital beamforming methods^8^. This balanced architecture provides sufficient flexibility for advanced multiplexing and multi-user strategies, while reducing hardware complexity and power consumption^9^.

In addition, it provides a higher sum rate than analog beamforming and approaches the performance of digital beamforming^10^. The design of hybrid beamforming requires decomposing the fully digital precoder into analog and digital precoders under strict hardware constraints such as unit-modulus phase shifters and coupling between analog and digital precoders in the signal-to-interference-plus-noise ratio (SINR) expression, resulting in a bilinear structure that leads to a non-convex optimization problem. Existing approaches found optimal hybrid beamforming solutions based on iterative algorithms, which solve the non-convex problem, such as the manifold optimization alternating minimization (MO-AltMin) algorithm, the semidefinite relaxation alternating minimization (SDR-AltMin) algorithm, and phase extraction alternating minimization (PE-AltMin)^11^, which first two use a MATLAB optimization software package such as Manopt^12^ and CVX^13^. For a fully connected structure, where all RF chains are connected to all antennas via phase shifters, the MO-AltMin algorithm alternately updates the digital beamforming using least squares and refines the analog beamforming through conjugate gradient descent on the manifold and repeats until convergence. This method achieves a near-optimal spectral efficiency, despite the high cost of complexity. On the other hand, the PE-AltMin algorithm reduced this complexity by enforcing the orthogonality constraint and updating the analog beamforming from simple phase extraction to an equivalent digital beamforming, which also significantly improves performance. For the partially connected structure, in which each RF chain is connected to a subset of antennas, the SDR-AltMin algorithm is developed, which exploits the block-diagonal structure of the analog beamforming and provides closed-form phase updates and optimal digital beamforming via convex optimization, and makes a significant improvement over only analog beamforming. However, these existing iterative approaches for hybrid beamforming face limitations of prohibitive computational complexity and excessive latency. Therefore, they cannot satisfy the demanding real-time needs of vehicular communications because they fail to address the requirements of latency-sensitive mission-critical communications^14^.

Deep learning (DL) has emerged as a promising paradigm for communication system applications because it differs from other iterative methods in behavioral applications^15^. In addition, it has a significant role in dealing with difficult problems in the physical layer^16^, including channel estimation^17^, global navigation satellite system (GNSS) jamming detection^18,19^, and beamforming^20–22^. However, solving complicated non-convex optimization problems that usually affect beamforming design is not the only achievement. On the other hand, DL models require a lot of offline training, and after training, they can make low-latency decisions during real-time operation owing to their powerful pattern recognition and regression capabilities. The main advantage of DL in beamforming is supposed to be apparent in 5G/6G cellular networks^23^, where instead of just overcoming the severe computational bottleneck limitations found in traditional algorithms, it also achieves real-time requirements in applications such as autonomous vehicular communications.

The application of DL in hybrid beamforming was introduced in^20^, which uses a convolutional neural network (CNN)-based beamforming design to map imperfect channel state information (CSI) matrices to the analog beamforming matrix trained by exhaustive labeled data from perfect CSI to obtain high spectral efficiency while reducing computational complexity for a single user. However, it cannot support multi-user beamforming where inter-user interference plays a major role and there is no enforcement to make sure of the unit-modulus constraint, which hinders the implementation of the analog beamforming matrix. Additionally,^21^ proposed a DL method that jointly selects antennas and hybrid beamforming for a single user by two CNN models, in which the first model selects the subarrays of antennas that maximize the spectral efficiency, and the second model predicts the analog and digital beamforming matrices. It was trained offline with exhaustive label data to be robust against imperfect CSI. Moreover, in^22^, a CNN-MIMO-based hybrid beamforming method is used in a multi-user mmWave massive MIMO system, where the model learns a mapping between the imperfect CSI and analog beamforming matrix in the transmitter and the receiver with perfect CSI label data. All of the aforementioned algorithms require a long training time, which complicates retraining when system configuration changes. Moreover, they are data-hungry in the training phase^22^, which demands a large memory allocation for storage. Additionally, they didn’t use realistic channel models, which may produce suboptimal beamforming matrices upon application in a practical scenario.

In this paper, the problem of designing hybrid beamforming in MU-MISO mmWave systems is addressed. Beyond this, scalability and durability are critical system-level considerations for large-scale or resource-constrained wireless deployments. Recent research has shown that abstraction-based modeling, such as geographical abstraction in mega-constellation networks^24^ and connectivity analysis in wireless-powered sensor networks^25^, can greatly improve scalability and robustness in large-scale and resource-constrained wireless networks at the system level. These perspectives strengthen the proposed DL-HBF framework’s applicability for large or resource-constrained deployments, where system resilience and observation-space abstraction are critical. The key contributions are:

- Structured procedures are developed to generate the supervised learning dataset. The input data consists of the angle, real and imaginary components of the channel matrices, while the output labels correspond to the indices of the optimal analog beamforming matrices. This dataset is generated based on DeepMIMO with a detailed configuration of environmental parameters, BS and user antenna arrays, and the specification of the number of users in MU-MISO mmWave systems.
- A DL-based hybrid beamforming method (DL-HBF) is proposed to design hybrid beamforming matrices in MU-MISO mmWave systems. It employs a CNN model to classify the optimal analog beamforming matrix directly from the input channel matrices. Subsequently, the digital beamforming matrix is calculated, enabling a complete hybrid beamforming design.
- A Comprehensive performance evaluation and robustness analysis of the proposed DL-HBF method and the traditional hybrid beamforming algorithms, such as MO-AltMin, PE-AltMin, SDR-AltMin, and orthogonal matching pursuit (OMP). This analysis employs a realistic ray-tracing-based mmWave channel model capturing practical propagation characteristics, demonstrating an acceptable sum rate while significantly reducing execution time and maintaining robustness under imperfect CSI—highlighting its real-world applicability and suitability for real-time deployments in future wireless systems.

**Notation**: Throughout this paper: $\textbf{A}$ is a matrix, $\textbf{a}$ is a vector, *a* is a scalar, and $\mathcal {A}$ is a tensor. $|\textbf{A}|$ is the determinant of $\textbf{A}$, where $\textbf{A}^\textsf{T}$, $\textbf{A}^\textsf{H}$, $\textbf{A}^*$ are its transpose, hermitian (conjugate transpose), and conjugate, respectively. $\textbf{I}_{K}$ is the identity matrix of dimension *K* and $\mathcal {C}\mathcal {N}(\textbf{m},\textbf{R})$ is a complex Gaussian random vector with mean $\textbf{m}$ and covariance $\textbf{R}$. For a matrix $\textbf{A}$, $[\textbf{A}]_{i,j}$ denotes the $(i,j)$th entry. Furthermore, $[\mathcal {A}]_{i,j,k,l}$ refers to the $(i,j,k,l)$-th element of a tensor $\mathcal {A}$. $\mathbb {E}\{\cdot \}$ denotes the statistical expectation and $\Vert \cdot \Vert _{\textrm{F}}$ is the Frobenius norm. The notation $(\cdot )^{\dagger }$ denotes the Moore-Penrose pseudo-inverse, while $\angle \{\cdot \}$ denotes the angle of a complex scalar/vector/matrix. The operator $\Re \{\cdot \}$ denotes the real part, while $\Im \{\cdot \}$ denotes the imaginary part of a complex quantity. The symbol $\odot$ is the Hadamard product, and $*$ is the convolution operation.

## System and channel models

**Fig. 1:**
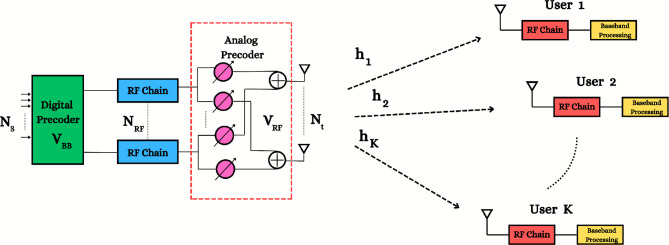
A block diagram of MU-MISO system with hybrid beamforming architecture at BS and a single antenna K users.

A narrowband MU-MISO mmWave downlink system with a fully connected hybrid beamforming architecture is considered, as shown in Fig. 1. The BS is equipped with $N_\textrm{t}$ antennas and $N_\textrm{RF}$ RF chains. Without loss of generality, we assume each user needs only a single stream. Because the total number of streams, $N_\textrm{s}$, equals the number of users, *K*, thus ${K} \le N_\textrm{RF} \le N_\textrm{t}$, and this affects the maximum number of users, which are limited by the number of RF chains. In the hybrid downlink beamforming architecture, the BS processes the data streams in baseband using a digital precoder, $\textbf{V}_\textrm{BB} = \left[ \textbf{v}_\mathrm {BB_1},..., \textbf{v}_{\textrm{BB}_K}\right] \in \mathbb {C}^{N_\textrm{RF} \times K}$, such that $\textbf{v}_{\textrm{BB}_k}$ is associated as a digital precoder for the data stream, which is intended for each user to transmit the symbol vector $\textbf{s} = \left[ s_\textrm{1},...,s_{K}\right] ^\textsf{H} \in \mathbb {C}^{{K}}$. That is satisfying $\mathbb {E}\{\textbf{s}\textbf{s}^\textsf{H}\} = \frac{P}{K} \textbf{I}_{K}$, where *P* is the total average transmitted power, under the assumption of equal power allocation across different users’ streams. Subsequently, the analog precoder $\textbf{V}_\textrm{RF} \in \mathbb {C}^{N_\textrm{t} \times N_\textrm{RF}}$, is implemented via analog phase shifters where $|\textbf{V}_{\text {RF}}(i,j)|^2 = 1, \quad \forall i,j$, which governs the direction of transmission across the $N_\textrm{t}$ antenna elements. The transmitted signal $\textbf{x}$ can be constructed by:

1 $$\begin{aligned} \textbf{x} = \textbf{V}_{\text {RF}} \textbf{V}_{\textrm{BB}} \textbf{s} = \sum _{k=1}^{K} \textbf{V}_{\textrm{RF}} \textbf{v}_{\textrm{BB}_k} s_k, \end{aligned}$$

where $\textbf{x} \in \mathbb {C}^{N_\textrm{t}}$ and $s_k$ is the transmitted symbol for the $k^{th}$ user. Consequently, the received signal of the $k^{th}$ user is $y_k$:

2 $$\begin{aligned} y_k = \underbrace{ \textbf{h}_k^\textsf{H} \textbf{V}_{\text {RF}} \textbf{v}_{\textrm{BB}_k} s_k}_{\text {desired signals}} + \underbrace{ \textbf{h}_k^\textsf{H} \sum _{\ell \ne k} \textbf{V}_\textrm{RF}\textbf{v}_{BB_\ell } s_\ell }_{\text {interference signals}} + \underbrace{ n_k}_{\text { noise}}, \end{aligned}$$

where $\textbf{h}_k \in \mathbb {C}^{N_\textrm{t}}$ is the channel vector between BS and the $k^{th}$ user and $n_k$ is a complex number that denotes the complex additive white Gaussian noise (AWGN) with $n_k \sim \mathcal {C}\mathcal {N} \left( 0,\sigma ^2\right)$.

Here, a flat-fading, narrow-band geometric mmWave channel model is considered with limited scattering. Therefore, we assume that the channel vector $\textbf{h}_k$ has $\textrm{L}$ scattering paths. Taking into account that we use a uniform linear array (ULA), therefore the channel vector can be expressed as^8^:

3 $$\begin{aligned} \textbf{h}_k = \sqrt{ \frac{N_{t}}{L_{k}} }\ \sum _{l=1}^{L_k} \alpha _{k,l} \textbf{a}_t(\theta _{k,l}), \end{aligned}$$

where $L_{k}$ is the number of scattering paths for the $k^{th}$ user, $\alpha _{k,l} \sim \mathcal{C}\mathcal{N}(0, 1)$ is the complex gain for the $l^{th}$ path for the $k^{th}$ user, $\theta _{k,l}$ is the angle of departure (AoD) of the $l^{th}$ path for the $k^{th}$ user and $\textbf{a}_t(\theta _{k,l}) \in \mathbb {C}^{N_\textrm{t}}$ is the antenna array response vector of the transmitter at the BS, which is given by:

4 $$\begin{aligned} \textbf{a}_t(\theta _{k,l}) = \frac{1}{\sqrt{N_t}} \begin{bmatrix} 1\\ e^{j \frac{2\pi }{\lambda } d \sin (\theta _{k,l})}\\ \vdots \\ e^{j \frac{2\pi }{\lambda } d (N_t-1) \sin (\theta _{k,l})} \end{bmatrix}, \end{aligned}$$

where $\lambda$ is the wavelength of the signal and $d$ is the antenna spacing.

## Problem formulation

For vehicular communications to serve high-throughput and latency-sensitive applications, real-time data interchange between automobiles and BS must be supported. As a result, the hybrid beamforming architecture of the MU-MISO system is required to optimize the sum rate, which can be defined as^9^:

5 $$\begin{aligned} \begin{aligned}&\underset{\textbf{V}_\textrm{RF},\, \textbf{V}_\textrm{BB}}{\text {max}} & R_{\text {sum}} = \sum _{k=1}^K \log _2 \left( 1 + \text {SINR}_k \right) , \\&\text {subject to} & \left\| \textbf{V}_\textrm{RF} \textbf{V}_\textrm{BB} \right\| _F^2 = K, \\ & &\left| [\textbf{V}_\textrm{RF}]_{i,j}\right| = 1,\quad \forall i,j \end{aligned} \end{aligned}$$

where the SINR for the $k^{th}$ user is:

6 $$\begin{aligned} \text {SINR}_k = \frac{\frac{P}{K}|\textbf{h}_k^\textsf{H} \textbf{V}_\textrm{RF} \textbf{v}_{\textrm{BB}_k} |^2}{\frac{P}{K}\sum \limits _{j \ne k} | \textbf{h}_k^\textsf{H} \textbf{V}_\textrm{RF} \textbf{v}_{\textrm{BB}_j} |^2 + \sigma ^2}. \end{aligned}$$

In this manuscript, the main objective is to find the optimal matrix $\textbf{V}_\textrm{RF}$ and $\textbf{V}_\textrm{BB}$, which maximizes the sum rate. This problem poses some challenges: firstly, it is a nonconvex problem because of the coupled variables $\textbf{V}_\textrm{RF}$ and $\textbf{V}_\textrm{BB}$, which result in bilinear and unit modulus constraints. Secondly, interference-dominant environments for low SINR, and finally, dynamic conditions for users that need real-time adaptability.

The optimal analog and digital beamforming matrices—in terms of maximizing the sum rate – can be obtained using a variety of conventional methods, including manifold optimization, successive convex approximation, alternating optimization, and semidefinite relaxation^11^. Unfortunately, most of these methods are computationally complex and are unable to adapt in real time to changing conditions^11^. DL, therefore, has a promising potential for solving this issue.

## Dataset generation

Obtaining the dataset is the first step in training DNNs. This dataset can be gathered for real deployments by the BS using real-time channel readings^26^. But in this instance, the system model is used to simulate the training dataset as mentioned in the previous section.

### Channel generation

First, we use the DeepMIMO framework^27^ to generate a suitable dataset for the MU-MISO mmWave system. This dataset is parameterized, which can be used in various configurations of wireless systems with the ability to adjust the channel parameters and represents environmental channels. It is generated with the aid of the Wireless InSite ray-tracing simulator and validated by channel measurements^28^. By specifying a particular simulation scenario, the realistic channel model is produced using the MU-MIMO system model. The active BS, the group of chosen users, the antenna arrangement at the BS and users, and the pertinent bandwidth are all important parameters of this simulation. Thus, each system and environmental characteristics are required to generate the appropriate channel impulse response and accurately mimic the required propagation environment.

### Input data

The input channel matrix $\textbf{H}$ for the *K* users is constructed as follows:

7 $$\begin{aligned} \textbf{H} = \begin{bmatrix} \textbf{h}_1&\textbf{h}_2&\cdots&\textbf{h}_K \end{bmatrix}^\textsf{H} \in \mathbb {C}^{K \times N_t}. \end{aligned}$$

where $\textbf{h}_k$ is the channel impulse response vector of the $k^{th}$ user.

Three matrices are then obtained from $\textbf{H}$ forming $\textbf{X}_A,\textbf{X}_R,\textbf{X}_I$. The first matrix is $[\textbf{X}_A]_{i,j} =\angle [\textbf{H}]_{i,j}$, which is the angle of the channel matrix elements. The second and third matrices are the real and imaginary parts of $\textbf{H}$, i.e., $[\textbf{X}_R]_{i,j} = \Re \{[\textbf{H}]_{i,j}\} \quad \text {and} \quad [\textbf{X}_I]_{i,j} = \Im \{[\textbf{H}]_{i,j}\}$. This representation enables the CNN model to accurately capture the spatial structure of the complex-valued channel impulse response. Therefore, the model gains direct access to the linear components of the channel by providing both the real and imaginary parts. In addition, the inclusion of phase information, formulated via the nonlinear arctangent relationship of the real and imaginary components, enhances the model’s ability to capture the inherent nonlinearity of the channel structure^29^. Furthermore, through simulation results, which will be shown shortly in the result section , we noticed that this representation demonstrates a notable improvement in the model’s accuracy.

### Output data

According to Algorithm 1, to generate the training labels required for the proposed DL-HBF method, an offline exhaustive search explored all feasible analog beamforming matrices in a predefined codebook $\mathcal {V} = \{ \textbf{V}_{\text {RF}}^{(1)}, \textbf{V}_{\text {RF}}^{(2)}, \ldots , \textbf{V}_{\text {RF}}^{(N_{cb})} \}$ where $N_{cb}$ is the number of codewords that maximize the sum rate of our system model. The analog beamforming codebook $\mathcal {V}$ is constructed based on the discrete Fourier transform (DFT) matrix $\textbf{U} \in \mathbb {C}^{N_t \times N_t}$^30^, which is defined as:

8 $$\begin{aligned} [\textbf{U}]_{s,c} = \frac{1}{\sqrt{N_t}} e^{-j 2\pi (s-1)(c-1)/N_t}, \quad 1 \le s,c \le N_t, \end{aligned}$$

where each column vector $\textbf{v}$ of $\textbf{U}$ is orthogonal and represents beams for analog beamforming matrix $\textbf{V}_{\text {RF}}$. The codebook is generated by selecting unique combinations of $N_{\text {RF}}$ columns from $\textbf{U}$, yielding the analog beamforming matrix:

9 $$\begin{aligned} \textbf{V}_{\text {RF}}^{(b)} = \left[ \textbf{v}_{1}^{(b)}, \ldots , \textbf{v}_{N_{\text {RF}}}^{(b)} \right] , \quad b = 1, \ldots , N_{\text {cb}}. \end{aligned}$$

Then it is normalized:

10 $$\begin{aligned} \textbf{V}_{\text {RF}}^{(b)} = \frac{\textbf{V}_{\text {RF}}^{(b)}}{\Vert \textbf{V}_{\text {RF}}^{(b)}\Vert _F}. \end{aligned}$$

Given a channel matrix $\textbf{H}$, we select the analog beamforming matrix, $\textbf{V}_{\text {RF}}$, from the codebook that maximizes the sum rate and compute the optimal digital precoder $\textbf{V}_{\text {BB}}$. Assuming $\textbf{H}_{\text {eff}}= \textbf{H} \textbf{V}_{\text {RF}}$ is the effective channel matrix and using minimum mean square error (MMSE)^31^:

11 $$\begin{aligned} \textbf{V}_{\text {BB}} =( \textbf{H}_{\text {eff}}^\textsf{H} \textbf{H}_{\text {eff}} + \frac{1}{\text {SNR}} \textbf{I}_{K} )^{-1} \textbf{H}_{\text {eff}}, \end{aligned}$$

where SNR is the signal-to-noise ratio. By normalizing $\textbf{v}_{\textrm{BB}_k}$, such that $\textbf{v}_{\textrm{BB}_k} = \frac{\textbf{v}_{\textrm{BB}_k}}{\Vert \textbf{V}_{\text {RF}} \textbf{v}_{\textrm{BB}_k} \Vert _F}$. To sum up, the output label data is calculated by specifying the index of the optimal analog beamforming matrix in the codebook, which leads to maximum sum rate due to Algorithm 1, and then digital beamforming is calculated by (11). Training and testing data are constructed from input data and output data mentioned previously to combine them as follows:

12 $$\begin{aligned} \mathcal {D}_{N_{\text {i}}} = \left( \left( \mathcal {X}^{(1)}, {z}^{(1)} \right) , \dots , \left( \mathcal {X}^{(N_{\text {i}})}, {z}^{(N_{\text {i}})} \right) \right) , \end{aligned}$$

and

13 $$\begin{aligned} \mathcal {D}_{\text {M}} = \left( \left( \mathcal {X}^{(1)}, {z}^{(1)} \right) , \dots , \left( \mathcal {X}^{(M)}, {z}^{(M)} \right) \right) , \end{aligned}$$

where $\mathcal {X}^{(N_{\text {i}})} \in \mathbb {R}^{K \times N_{t} \times 3}$ is the input data for each $N_{\text {i}}$ sample, ${z}^{(N_{\text {i}})}$ is the index of the optimal analog beamforming matrix $\textbf{V}_\text {RF}$ class for $N_{\text {i}}$ sample, $\mathcal {D}_{N_{\text {i}}}$ is the training dataset, $N_{\text {i}}$ is the number of samples in the dataset which is divided into training and validation, $\mathcal {D}_{\text {M}}$ is the testing dataset, and $\text {M}$ is number of samples in the test dataset.

**Fig. 2:**
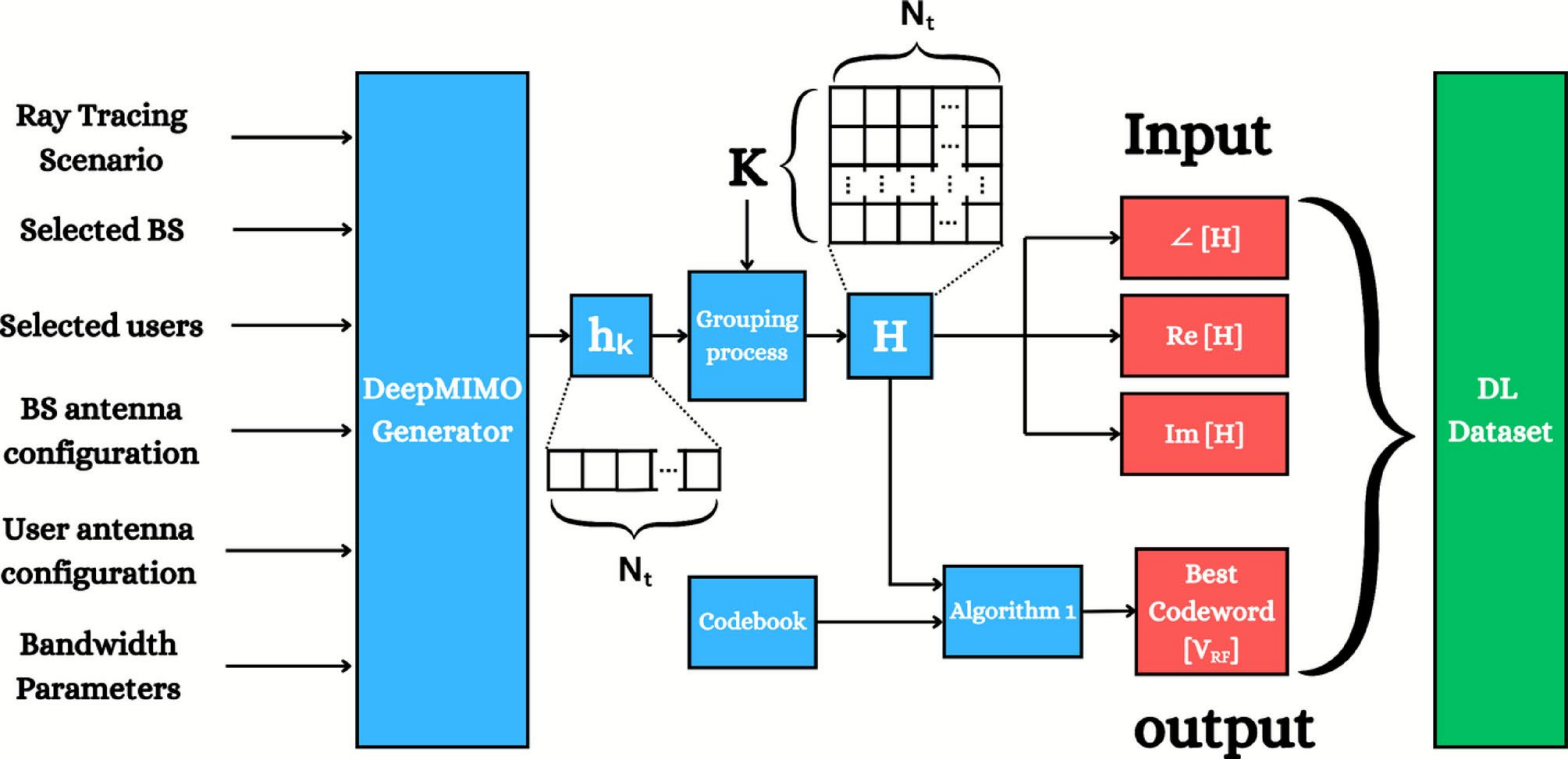
A block diagram of the procedures of dataset generation.

Based on the procedures illustrated in Fig. 2, the dataset can be generated as follows. **Firstly**, specifying the raytracing scenario that shows the BS’s location, the users’ positions geographically distributed in a certain outdoor environment, BS and users are selected, design the BS and users’ antenna configurations (how many antennas, and antenna spacing and rotation) and system bandwidth then the DeepMIMO generator is used to generate channel parameters (angles of arrival/departure, path gains, $\cdots$ etc.) between the BS and users. **Secondly,** we specify how many users are assumed in our case of the MU-MISO system to group the new channel matrix as in (7). **Thirdly**, the output will have two paths, first path is to create the input data with $\angle [\textbf{H}], \Re \{[\textbf{H}\}, \Im \{[\textbf{H}\}$ and the second path will be input to algorithm 1 to select the optimal analog beamforming matrix (codeword) that maximizes the sum rate of the MU-MISO system according to Algorithm 1. **Finally**, the input and output data will create the dataset needed for training the proposed DL-HBF method.

**Algorithm 1:**
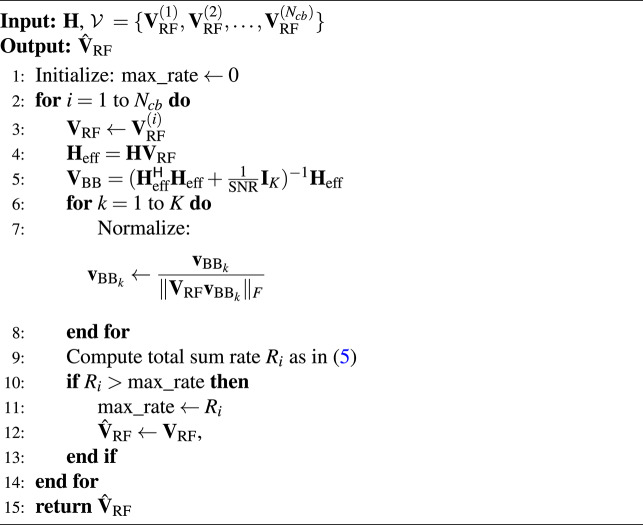
Hybrid Precoding for MU-MISO

## The proposed DL-HBF method

**Fig. 3:**
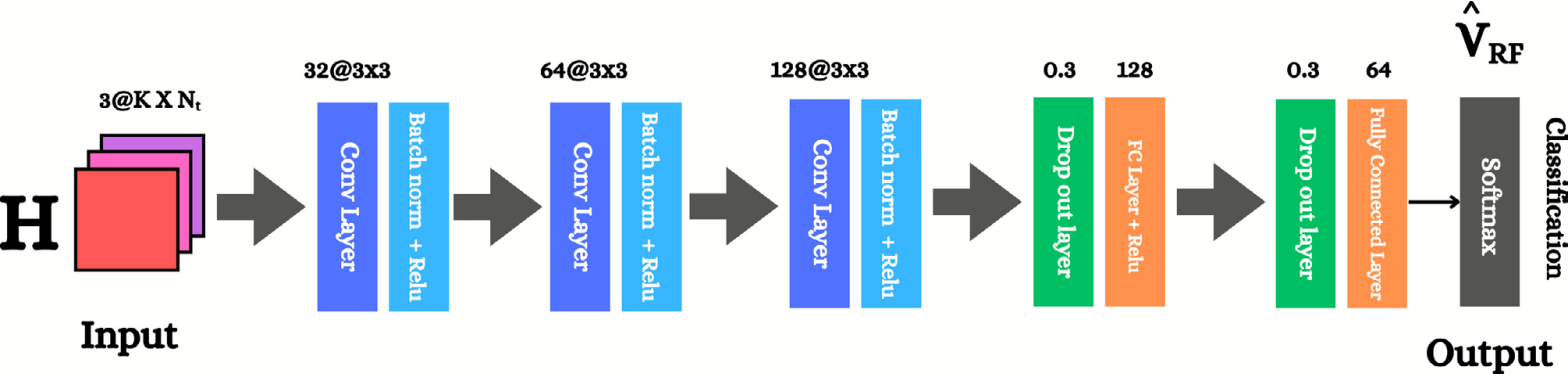
A block diagram of the proposed DL model. The model depends on the input of (Angle, Real, Imag) channel tensor $\mathcal {X}$ to predict the class of optimal analog beamforming matrix.

To address the limitations mentioned in the proplem formulation section, we propose a DL-HBF method to obtain the optimal analog and digital beamforming matrices that maximize the sum rate based on DL. The objective of the CNN model shown in Fig. 3 is to classify the input data $\mathcal {X}$ into the index of the optimal analog beamforming matrix $\textbf{V}_\text {RF}$ selected from the codebook $\mathcal {V}$. This approach will be illustrated in the upcoming subsections.

### Input representation and normalization

The input data $\mathcal {X}^{(N_{\text {i}})}$, which has encapsulated features and characteristics of the channel between the BS and users is divided into $\angle [\textbf{H}], \Re \{\textbf{H}\}, \Im \{\textbf{H}\}$ angle, real part and imaginary part of the channel matrix, respectively. To enhance numerical stability and speed up convergence, the Z-score normalization method will be used to normalize the input features along the sample axis, which enforces a zero mean and a variance of unity. Input elements $[\mathcal {X}^{(N_{\text {i}})}]_{i,j,c}$ are normalized by Z-score:

14a $$\begin{aligned} \left[ \mathcal {X}^{(N_{\text {i}})}\right] _{i,j,c}^{\text {norm}}&=\frac{\left[ \mathcal {X}^{(N_{\text {i}})}\right] _{i,j,c}-\mu _{i,j,c}}{\sigma _{i,j,c}+\epsilon },\end{aligned}$$

14b $$\begin{aligned} \mu _{i,j,c}&= \frac{1}{N_i} \sum _{n=1}^{N_i} \left[ \mathcal {X}^{(n)}\right] _{i,j,c}\end{aligned}$$

14c $$\begin{aligned} \sigma _{i,j,c}&= \sqrt{ \frac{1}{N_i} \sum _{n=1}^{N_i} \left( \left[ \mathcal {X}^{(n)}\right] _{i,j,c} - \mu _{i,j,c} \right) ^2 } \end{aligned}$$

where $\mu _{i,j,c}$ is the mean of the input data, $\sigma _{i,j,c}$ is the standard deviation of the input data across the sample dimension $N_{\text {i}}$ for each element (*i*, *j*, *c*) and $\epsilon$ is a small constant to avoid division by zero.

### Model architecture

A convolutional neural network is designed in order to deal effectively with the spatial and temporal characteristics and features of the CSI. As shown in Fig. 3, the designed architecture contains three consecutive convolutional blocks, which perform hierarchical feature extraction and then connect to fully connected layers that reduce dimensionality before ending with a Softmax activation function (output layer) for multi-class classification.

- **Convolutional layer** The hierarchical feature extraction ability of CNNs learn very particular kinds of local spatial patterns and combines them to form increasingly abstract features that will help optimize beamforming matrices.
  15 $$\begin{aligned} \begin{aligned} \left[ \mathcal {Z}^{(k)}\right] _{i,j,n} =&\sum _{c=1}^{D_{in}} \sum _{u=1}^{R} \sum _{v=1}^{R} \left[ \mathcal {A}^{(k-1)}\right] _{i+u-1, j+v-1, c} \cdot \left[ \mathcal {W}^{(k)}\right] _{u,v,c,n} + \textbf{b}_{n}^{(\text {k})}, \end{aligned} \end{aligned}$$
  where $\mathcal {Z}^{(k)} \in \mathbb {R}^{K \times N_{t} \times D_{out}}$ is the output of the convolutional layer, $D_{out}$ is the output depth, $\mathcal {W}^{(k)} \in \mathbb {R}^{R \times R \times D_{in} \times D_{out}}$ is the filter weights of $k^{th}$ layer, each filter has a spatial dimention of $R \times R$ which is a learnable weight matrix that slides over the input data and performs the convolutional operation to extract features and spatial pattern, $D_{in}$ is the input depth, $\mathcal {A}^{(k-1)} \in \mathbb {R}^{K \times N_{t} \times D_{in}}$ is the output of previous activation layer, $\mathcal {A}^{(0)}=\mathcal {X}^{\text {norm}}$, besides, it is the input to the $k^{th}$ layer and $\textbf{b}_{n}^{(k)} \in \mathbb {R}^{D_{out}}$ is the bias term of $k^{th}$ layer. There are three convolutional layers with increasingly larger feature maps: $\mathcal {W}^{(1)}$ has 32 filters with size 3$\times$3, $\mathcal {W}^{(2)}$ has 64 filters of size 3$\times$3, and $\mathcal {W}^{(3)}$ has 128 filters of size 3$\times$3. The three layers have the same filter size but double the channel depth at each stage to enable hierarchical feature learning as in^32^.
- **Batch normalization layer and activation function** It normalizes the activations of a layer in a mini-batch, stabilizes, accelerates the training process, and reduces internal covariate shift by allowing each layer to learn from inputs of a fixed distribution^33^.
  16 $$\begin{aligned} \left[ \mathcal {Z}^{(k)}_{B}\right] _{i,j,n}^{\text {norm}} = \gamma ^{(k)}_n \left( \frac{[\mathcal {Z}^{(k)}_{B}]_{i,j,n} - \mu _{\textbf{n}}^{(k)}}{\sqrt{(\sigma ^2)_{\textbf{n}}^{(k)} + \epsilon }} \right) + \beta ^{(k)}_n , \end{aligned}$$
  where $[\mathcal {Z}^{(k)}_{B}]^{\text {norm}}$ is the normalized output of the $k^{th}$ batch normalization layer for mini-batch size per depth *n*, $\mathcal {Z}^{(k-1)}_{B}$ is the input to the batch normalization layer for mini-batch size of depth *n*, $\mu _{n}^{(k)}$, $(\sigma ^2)_{n}^{(k)}$ are the mean and variance of the $k^{th}$ batch normalization layer, which is computed per depth n over mini-batch size, respectively, and $\gamma ^{(k)}_n$, $\beta ^{(k)}_n$ are learnable scale and shift parameters for depth n in the $k^{th}$ batch normalization layer. Rectified linear unit (ReLU) activation is used as an activation function, which adds non-linearity to the model so that it has the capability to learn complex mappings between input and output, and is less computationally expensive than tanh and sigmoid functions.
  17 $$\begin{aligned} \left[ \mathcal {A}_{B}^{(k)}\right] _{i,j,n} = \max \left( 0, \left[ \mathcal {Z}_{B}^{(k)}\right] _{i,j,n}^{\text {norm}}\right) , \end{aligned}$$
  where $[\mathcal {Z}^{(k-1)}_{B}]^{\text {norm}}$ is the ReLU activation input at the $k^{th}$ layer and $\mathcal {A}_{B}^{(k)}$ is the ReLU activation output in the $k^{th}$ layer.
- **Fully connected layer** Upon feature extraction via convolutional layers, fully connected layers are then used for projecting the learned features to the output, and they help integrate information from different parts of the input and then make the final choice.
  18 $$\begin{aligned} \textbf{u}^{(\text {k})} = \textbf{F}^{(\text {k})}\textbf{o}^{(\text {k-1})} + \textbf{d}^{(\text {k})}, \end{aligned}$$
  where $\textbf{u}^{(\text {k})} \in \mathbb {R}^{Q}$ is the output of the $k^{\text {th}}$ fully connected layer, $\textbf{o}^{(k-1)} \in \mathbb {R}^{G}$ is the input for the fully connected layer which is flattened and *G* is total number of elements after flattening and dropout, $\textbf{d}^{(\text {k})} \in \mathbb {R}^{Q}$ is the bias term of $k^{\text {th}}$ fully connected layer and $\textbf{F}^{(k)} \in \mathbb {R}^{Q \times G}$ is the associated weights of the $k^{th}$ fully connected layer.
- **Dropout layer** To minimize overfitting, dropout regularization is applied during the training process when a fraction p of the neuronal units is randomly shut down with probability p at each training iteration, forcing the network to learn robust features. The model uses a dropout rate of p = 0.3^34^.
  19 $$\begin{aligned} \textbf{u}_{\text {dropout}}^{(k)} = \textbf{m}^{(k)} \odot \textbf{u}^{(k)}. \quad m_{i} \sim \text {Bernoulli}(1-p) \end{aligned}$$
- **Softmax activation function (Output Layer)** It’s the final step for multi-class classification, and Softmax converts the raw output scores (logits) into probabilities, which are defined as
  20 $$\begin{aligned} {P}({u}_i) = \frac{e^{u_i}}{\sum _{j=1}^{N_{cb}} e^{u_j}}, \quad i= 1,\dots ,N_{cb} \end{aligned}$$
  where $u_i$ is the raw score for class *i*, $N_{cb}$ is the number of classes, which equals 64 in our case.

Subsequently, the optimal analog beamforming index can be obtained by selecting the index corresponding to the highest predicted probability calculated by the Softmax activation function, which is given by:

21 $$\begin{aligned} \hat{u} = \arg \max _{i} {P}({u}_i) \quad i= 1,\dots ,N_{cb} , \end{aligned}$$

where $\hat{u}$ denotes the index of the predicted codeword $\hat{\textbf{V}}_\text {RF}$.

### Training and loss function

Cross-entropy loss $\mathcal {L}$, which has a probabilistic foundation and provides smooth gradients, making it suitable for multi-class classification tasks, is used to train the classification model. The objective is to minimize this loss by adjusting the weights of the network during the training phase as follows^35^:

22 $$\begin{aligned} \mathcal {L} = -\sum _{i=1}^{N_{cb}} \bar{u}_{i} \log {P}({u}_{i}), \end{aligned}$$

where $\bar{u}_{i} \in \{0,1\}$ is the one-hot encoded ground-truth label for class *i* and ${P}({u}_{i})$ is the predicted probability for class *i*.

The adaptive moment estimation (Adam) optimizer is used to optimize the model parameters because of its efficient handling of sparse gradients, adaptability to different learning rates, and better convergence rate.

23 $$\begin{aligned} \theta _{t+1} = \theta _{t} - \eta \frac{\hat{{m}}_{t}}{\sqrt{\hat{{v}}_{t}} + \epsilon }, \end{aligned}$$

where $\theta _{t}$ denotes an individual model parameter (weight or bias) in the time step *t*, $\eta$ is the learning rate, $\hat{{m}}_t$ is the bias-corrected first moment estimate and $\hat{{v}}_t$ is the bias-corrected second moment estimate.

**Fig. 4:**
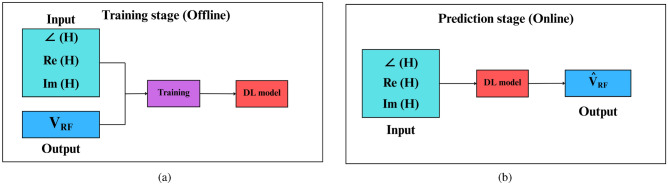
The training and prediction phases of the proposed DL-HBF model.

The CNN model has two stages as shown in Fig. 4. The first stage (training stage), where input channel tensor including $\angle [\textbf{H}], \Re \{[\textbf{H}\}, \Im \{[\textbf{H}\}$ and optimal analog beamforming $\textbf{V}_{\text {RF}}$ codewords are used to train the model. After training the CNN model, the second stage will appear (the classification or online stage), which will classify the optimal analog beamforming matrix $\textbf{V}_{\text {RF}}$ then the digital beamforming matrix $\textbf{V}_{\text {BB}}$ will be calculated as mentioned before in the output data subsection.

## Results and discussion

In this section, the performance of the proposed DL-HBF method is evaluated and compared with other benchmark methods in the MU-MISO system, including fully digital MMSE, MO-AltMin, SDR-AltMin, and PE-AltMin algorithms. Here, an MU-MISO downlink transmission system is considered with a BS that has a fully connected hybrid beamforming architecture with transmitting antennas $N_\textrm{t} = 8$, RF chains $N_\textrm{RF} = 4$, $N_\textrm{s}=4$, and every user has a single stream of data. The BS serves ${K} = 4$ users with a single antenna. We implement the proposed DL-HBF algorithms using MATLAB R2024a on a computer with an E5-1650 V3 CPU, 32 GB RAM, and an Nvidia Quadro K2200 4 GB GPU.

### Environment setup

**Fig. 5:**
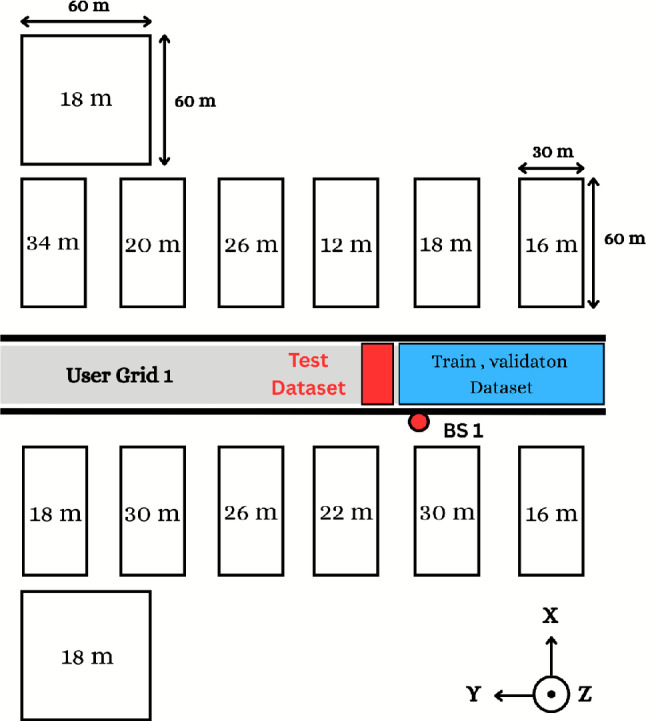
The top view of environment setup in which BS serves users located in the user grid 1, highlighting their locations and regions for training and testing datasets.

As shown in Fig. 5, an mmWave system is adopted, where the BS simultaneously serves multiple users over 60 GHz. A DeepMIMO scenario (O1_60), which shows the environment geometry, is used where:

- **Site plan**: The grid for users is in a 300 m long and 40 m wide street, the heights of all buildings are indicated, the buildings along the street have bases with 30 m x 60 m dimensions and two other far buildings with bases 60 m x 60 m dimensions, and a 60 GHz 3-layer dielectric material for the buildings, as shown on Fig. 5. A 60 GHz single-layer dielectric for the ground proves the importance of ray-tracing parameters such as reflection and penetration coefficients that accurately describe the model of the mmWave system. Each channel can undergo a maximum of 4 reflections before reaching the receiver.
- **BS**: BS 1 is 6 m in height, it has a ULA with 8 antennas where each antenna is an isotropic antenna. They use 30 dBm transmitter power, half-wavelength antenna spacing, and the bandwidth is 50 MHz.
- **Users**: The user has a single isotropic antenna at a height of 2 m. Users are uniformly distributed in the user grid (UG1) in the main street with a length of 550 m and a width of 35 m, has 2751 rows, and each row has 181 users and 20 cm spacing between adjacent users.

### Model evaluation and tuning

Here, we evaluate the CNN model by calculating the accuracy, precision, recall, and F1-score, respectively:

- Accuracy: which directly indicates how the model predicts the right index of classes.
  24 $$\begin{aligned} \begin{aligned} \text {Accuracy}=&\left( \frac{\text {Number of Correct Predictions}}{\text {Total Number of Predictions}} \right) \times 100 \end{aligned} \end{aligned}$$
- Precision: which provides the percentage of correct positive predictions among all positive predictions.
  25 $$\begin{aligned} \text {Precision}_i = \frac{TP_i}{TP_i + FP_i} \end{aligned}$$
- Recall (sensitivity): which provides the percentage of correct positive predictions among all actual positives.
  26 $$\begin{aligned} \text {Recall}_i = \frac{TP_i}{TP_i + FN_i} \end{aligned}$$
- F1-score: which combines precision and recall in one metric and avoids the model when it is very selective (high precision with low recall) or catches many positives but with wrong ones (high recall with low precision).
  27 $$\begin{aligned} \text {F1-score}_i = 2 \times \frac{\text {Precision}_i \times \text {Recall}_i}{\text {Precision}_i + \text {Recall}_i} \end{aligned}$$

where *i* denotes the class index, *TP* is true positive, *FP* is false positive and *FN* is false negative. After the parameters are selected as input to the DeepMIMO generator as mentioned before, an output channel is generated for every user, then a new channel matrix is created by combining 4 users’ channels and Algorithm 1 is used to generate the dataset as shown in Fig. 2. Users from “active_user_first” = 1 to “active_user_last” = 560 were used for the first dataset with 10000 samples. The dataset is split into 80% for training and 20% for validation. The second dataset, which is dedicated to testing, uses users from “active_user_first” = 561 to “active_user_last” = 610 and takes 2000 samples.

As shown in Table 1, the hyperparameters of our CNN model used in the proposed DL-HBF method are listed to obtain the optimal analog beamforming class.

**Table 1:** Proposed DL_HBF hyper-parameters.

Parameter	Set value
Mini-batch size	128
Initial learning rate	0.0001
Number of epochs	50
Optimizer	Adam
Dropout rate	0.3
Filter size	$3 \times 3$
Zero padding	1
Number of convolutional layers	3
Loss function	Cross-Entropy
Batch normalization momentum	0.99
Batch normalization $\epsilon$	$10^{-3}$

**Fig. 6:**
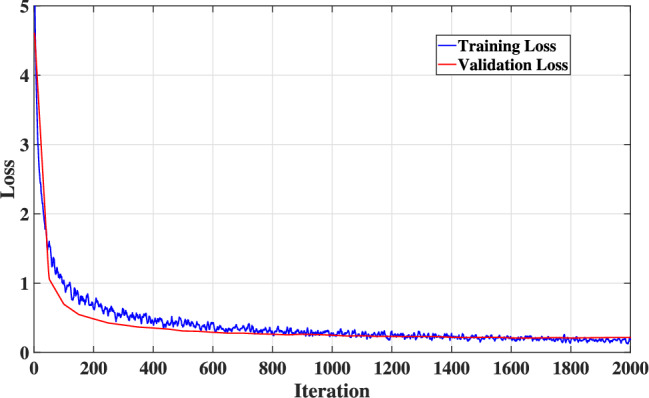
The model’s training and validation loss curves.

Figure 6 illustrates the learning curves, which demonstrate the critical insights of the stability and convergence behavior of the proposed DL-HBF model. As observed from Fig. 6, the training loss decreases steadily, which indicates that the model is learning from the training data. Moreover, the validation loss also decreases and closely follows the training loss, which indicates the model is generalizing well to unseen channel realizations and not significantly overfitting. Consequently, both curves appear to converge to a low stable value, which is a strong indicator of success and a stable training process.

As shown in Table 2, the proposed DL-HBF model achieves a classification accuracy of 93.2% in validation and 91.6% in testing, demonstrating strong learning and generalization capability. Additionally, it has high precision, which indicates that the model reliably predicts the correct analog beamforming class and effectively minimizes false selections, which is essential to avoid power leakage toward unintended directions. Despite the low recall values, this reflects the model’s conservative decision behavior, favoring precision to maintain stable performance. On the other hand, the F1-score confirms the trade-off between precision and recall.

**Table 2:** Performance metrics.

Metric	Validation dataset	Testing dataset
Accuracy (%)	93.2	91.6
Precision (%)	91.0	90.0
Recall (%)	79.0	71.0
F1 Score (%)	84.0	79.0

These metrics, while standard in classification tasks, are also indirectly related to hybrid beamforming performance. Specifically, accurate classification leads to the selection of analog beamforming matrices whose corresponding digital beamforming matrices maximize the achievable sum-rate.

**Fig. 7:**
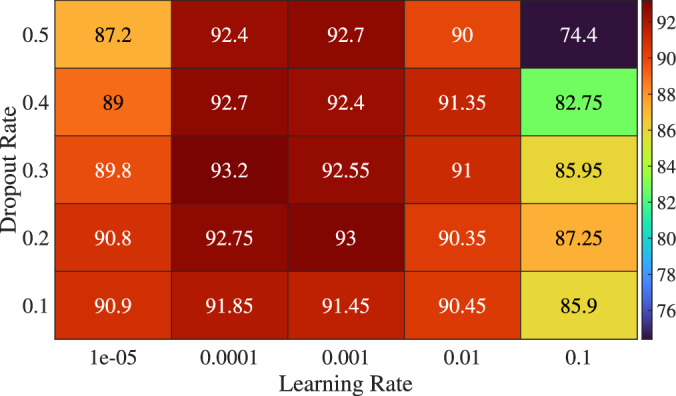
The model’s accuracy of different learning rates and dropout rates.

As shown in Fig. 7, which presents a comprehensive evaluation of how dropout rate and learning rate influence the accuracy of the proposed DL-HBF model. For the learning rate, the highest accuracy is obtained at learning rates 0.0001 and 0.001, as these values achieve the appropriate balance between convergence speed and stability. Conversely, the performance is degraded at a learning rate of 0.1, which indicates potential instability or divergence during training. The dropout rates of 0.2 and 0.3 yield a relatively high accuracy, which indicates effective regularization and improved performance. This aligns with the regularization theory in DL to mitigate overfitting and encourage the model to learn robust features that generalize effectively to unseen data. Additionally, it shows that the optimal dropout rate depends on the learning rate, and these accuracy values support the importance of tuning hyperparameters rather than optimizing them in isolation.

**Fig. 8:**
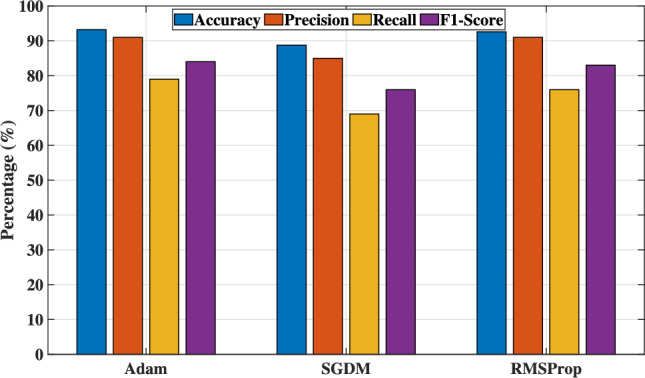
The model’s accuracy of different optimizers.

Figure 8 shows the different optimization techniques such as Adam, stochastic gradient descent with momentum (SGDM) and root mean square propagation (RMSProp) which provide a full picture of classification effectiveness by metrics’ evaluation such as *Accuracy*, *Precision*, *Recall* and $F1\_score$. Adam optimizer yields the best performance because it combines the advantages of the adaptive gradient algorithm (AdaGrad) and RMSProp, which uses adaptive learning rates and estimate the moment to speed up convergence and avoid local minima. On the other hand, the SGDM relies on the fixed learning rate and lacks adaptive gradient scaling, which results in the lowest performance. Furthermore, RMSProp provides intermediate performance that is better than SGDM due to its ability to adapt the learning rate and slightly lower than Adam, as it lacks the momentum terms. Overall, Adam optimizer proves most effective in capturing the nonlinear relationship of the input channel matrix and the optimal beamforming class, which highlights the importance of choosing the optimizer in DL models.

**Fig. 9:**
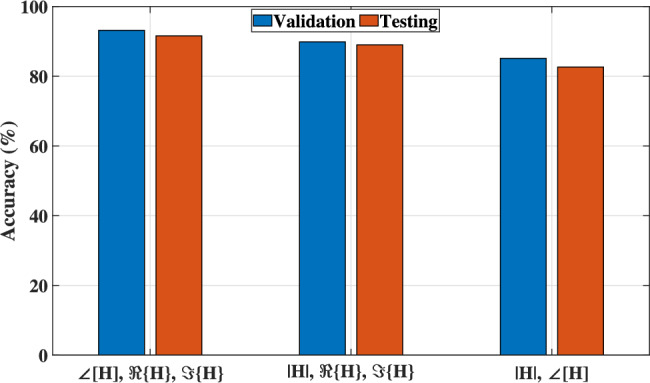
The model’s accuracy of different input representations.

The input channel tensor $\mathcal {X}$ is divided into different matrices, which will be the features of the model. As shown in Fig. 9, which illustrates that $\angle [\textbf{H}], \Re \{\textbf{H}\}, \Im \{\textbf{H}\}$ offers better accuracy than the other representations due to the $\left| \textbf{H} \right| , \angle [\textbf{H}]$ lack of directly providing real and imaginary, which reduced the ability of the model to capture complex spatial correlation in the input data. In addition, $\left| \textbf{H} \right| , \Re \{\textbf{H}\}, \Im \{\textbf{H}\}$ have redundant information, which potentially confuses the network, despite $\angle [\textbf{H}]$, which requires a nonlinear operation that is not easy for CNN to learn, and by providing it directly, it will be more effective.

The training time is a crucial aspect of the system’s overall efficiency and complexity in offline mode. The relevant metric to measure that is the number of trainable parameters and an indication of the complexity of the model in offline mode. From Table 3, which illustrates that the proposed model has fewer parameters, resulting in a reduced training overhead and shorter training time. To ensure objective comparison for different models, the same system configuration is used: $K=4$, $N_\text {RF}=4$, and $N_\text {t}=8$. Additionally, in^21^, the dataset generation time was approximately 2 hours and 2 minutes dedicated to this task to generate 10000 samples with the publicly available code and the author’s permission. However, our dataset generation required 53.7 seconds to generate the same samples. This comparison was implemented under the same system model and hardware setup mentioned before to ensure fairness. In contrast, this reduction in time is attributed to different data preparation methodologies. Our dataset relies on precomputed ray-tracing results generated by Recom Wireless InSite, which retrieved and processed depending on specific configurations, plus Algorithm 1 to construct the output data, reducing computational overhead. On the other hand, ^21^ generates the dataset from scratch, including random data generation, numerous nested loops to construct the channel matrix, and the optimization algorithm to construct output data, considering delay and computational complexity. Therefore, the time efficiency of our method proves especially advantageous in the training phase, including scalability and retraining new configurations and scenarios.

**Table 3:** Parameters in DL-based hybrid beamforming models.

Model	Number of parameters
Proposed DL-HBF	626,368
CNN-MIMO^22^	11,053,481
HBDL^20^	1,887,136
$\text {CNN}_{AS} + \text {CNN}_{RF}$^21^	951,226

### System performance

To evaluate the proposed model’s performance in terms of maximizing sum rate, it should be compared with traditional methods. Algorithms such as the MO-AltMin algorithm, the SDR-AltMin algorithm, and the PE-AltMin algorithm in^11^ and the OMP algorithm in^8^, we adopt these algorithms in the MU-MISO system to suit our multiple users case and apply them on the channel generated from DeepMIMO. These algorithms require a fully digital beamforming matrix as input to construct the analog and digital beamforming matrices, except the OMP algorithm, which additionally requires a predefined DFT codebook. The minimum mean square error (MMSE) is used as the optimal digital beamforming matrix needed for these traditional algorithms.

**Fig. 10:**
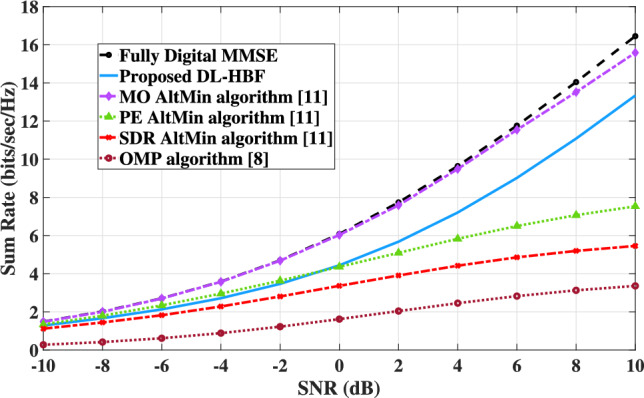
The sum rate of the proposed DL-HBF in MU-MISO system at $N_t = 8,\ N_{RF} = K = 4$.

Figure 10 illustrates that the DL-HBF method performs better than the PE-AltMIN, SDR-AltMin and OMP algorithms but less than the MO-AltMin algorithm in identifying the optimal hybrid beamforming matrices that maximize the sum rate in the MU-MISO system. However, the performance gap of the MO-AltMin algorithm is primarily attributed to advanced optimization techniques, including computationally intensive procedures such as the conjugate gradient algorithm on a Riemannian manifold to get the nearest optimal solutions. In contrast, the PE-AltMIN algorithm is a simpler algorithm but with limited performance due to enforcing an orthogonality constraint on the digital beamforming matrix. Additionally, the SDR-AltMin algorithm adopts a partially connected structure, which inherently reduces beamforming gains. For the OMP algorithm yields the lowest performance due to its ignorance of the joint effect of $N_{RF}$ columns which can’t capture interactions between RF chains, and also the lack of iterative refinement.

As shown in Table 4, the computational complexity of the proposed DL-HBF method is analyzed by considering its two stages. The first stage, the prediction of the analog beamforming matrix via the CNN model, is dominated by the convolution layer, which has a complexity of $\mathcal {O}(K N_\textrm{t} R^2 D_{in} D_{out})$. The second stage, the calculation of the digital beamforming matrix, involves several matrix operations, which is dominated by $\mathcal {O}(K^3)$. Therefore, the overall complexity of the proposed DL-HBF method is driven by these two stages, which is the forward pass that does not depend on $I_L$ iterations. Conversely, the traditional hybrid beamforming algorithms such as MO-AltMIN, PE-AlTMin, and SDR-AltMin are iterative in nature, and their computational complexity scales linearly with the number of iterations $I_L$. This highlights the competitive advantage of the proposed DL-HBF method at computational complexity in systems where low-latency beamforming is required. For the execution time of the system mentioned before, the proposed DL-HBF method will execute the optimal analog beamforming matrix and then calculate the digital beamforming matrix in 0.0042 s, the MO-AltMIN algorithm in 0.4397 s, the PE-AlTMin algorithm in 0.0022 s, the SDR-AltMin algorithm in 3.18774 s, and the OMP algorithm in 0.00025 s. Therefore, the SDR-AltMin algorithm is out of the comparison.

**Table 4:** The computational complexity of the proposed DL-HBF.

Method	Computational complexity
Proposed DL-HBF	$\mathcal {O}(K N_\textrm{t} R^2 D_{in} D_{out} + K^3)$
MO-AltMIN algorithm^11^	$\mathcal {O}(I_L (N_\textrm{t} N_\textrm{RF}^2 + N_\textrm{t} N_\textrm{RF} K))$
PE-AlTMin algorithm^11^	$\mathcal {O}(I_L (K N_\textrm{t} N_\textrm{RF} + K^2 N_\textrm{RF})$
SDR-AltMin algorithm^11^	$\mathcal {O}(I_L (K N_\textrm{RF})^6)$
OMP algorithm^8^	$\mathcal {O}(N_\textrm{RF} N_\textrm{cb} N_\textrm{t} K)$

**Fig. 11:**
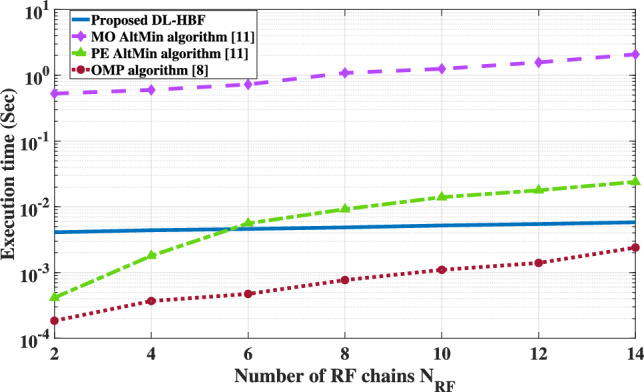
The execution time of the proposed DL-HBF in MU-MISO system at $N_t = 16$.

As shown in Fig. 11, which presents the average execution time of the proposed DL-HBF method, the MO-AltMIN, the PE-AlTMin and the OMP algorithms for different values of $N_{\text {RF}}$ and constant $N_\text {t}$ = 16. Results show that the MO-AltMIN algorithm has more execution time than others due to its exhaustive iterations, and this time increases significantly as $N_{\text {RF}}$ increases. However, the PE-AlTMin algorithm has less execution time than the proposed DL-HBF method in $N_{\text {RF}}$ = 2 and 4, it will take more execution time than the proposed DL-HBF method by increasing $N_{\text {RF}}$ and iterative updates. Additionally, the OMP algorithm achieves the lowest execution time due to its simplicity, but it increases proportionally to $N_{\text {RF}}$. This highlights the robustness and scalability of the proposed DL-HBF method, which maintains a nearly constant execution time as $N_{\text {RF}}$ increases. These merits underscore the potential of the proposed method for real-time applications.

The availability of perfect CSI is unrealistic due to estimation and feedback errors. To analyze the robustness of algorithms for imperfect CSI cases and evaluate their performance in terms of normalized mean square error (NMSE). The estimated channel matrix $\hat{\textbf{H}}$ can be expresed as:

28 $$\begin{aligned} \hat{\textbf{H}} = \textbf{H} + \textbf{E}, \end{aligned}$$

where $\textbf{E} \sim \mathcal{C}\mathcal{N}\left( \textbf{0}, \sigma _e^2 \textbf{I}\right)$ represents the channel estimation error matrix. Therefore, the degree of imperfection is characterised by:

29 $$\begin{aligned} \textrm{NMSE}_{\textbf{H}} = \frac{\Vert \hat{\textbf{H}} - \textbf{H}\Vert _F^2}{\Vert \textbf{H}\Vert _F^2}. \end{aligned}$$

**Fig. 12:**
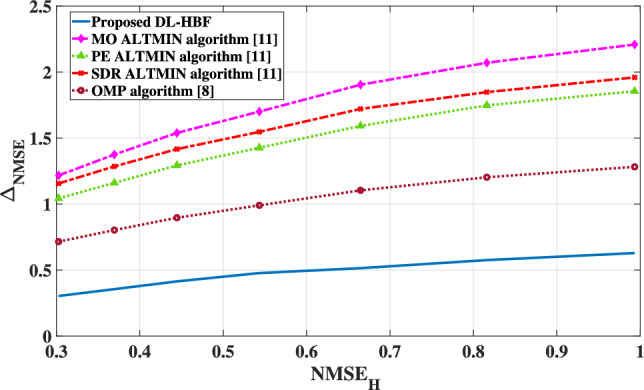
The degradation performance of the proposed DL-HBF under imperfect CSI.

To assess the approximation quality of hybrid beamforming matrices, NMSE is computed with respect to the optimal fully digital beamforming matrix by:

30 $$\begin{aligned} \textrm{NMSE}_{\textbf{V}} = \frac{\Vert \textbf{V}_{\textrm{RF}}\textbf{V}_{\textrm{BB}} - \textbf{V}_{\textrm{opt}}\Vert _F^2}{\Vert \textbf{V}_{\textrm{opt}}\Vert _F^2}, \end{aligned}$$

where $\textbf{V}_{\textrm{opt}}$ is the optimal fully digital beamforming matrix. When the hybrid beamforming is designed based on imperfect CSI instead of perfect CSI, its performance deteriorates. This degradation represented by the difference between the NMSE values obtained under imperfect and perfect CSI:

31 $$\begin{aligned} \Delta _{\textrm{NMSE}} = \textrm{NMSE}_{\mathbf {V_i}} - \textrm{NMSE}_{\mathbf {V_p}}, \end{aligned}$$

where $\textrm{NMSE}_{\mathbf {V_i}}$ calculated related to imperfect CSI and $\textrm{NMSE}_{\mathbf {V_p}}$ calculated related to perfect CSI. As shown in Fig. 12, all algorithms experience a degradation in performance as the CSI error increases. However, the extent of degradation varies significantly across algorithms. The proposed DL-HBF method consistently achieves the lowest $\Delta _{\textrm{NMSE}}$ across all $\textrm{NMSE}_{\textbf{H}}$, demonstrating superior robustness to channel estimation error due to its training on some noisy channels. On the other hand, other algorithms result in high degradation increases proportionally with estimated channel error. This confirms their reliance on perfect CSI and their limitations with noisy and imperfect CSI.

## Conclusion

In this paper, we presented a proposed DL-HBF method to get the optimal beamforming matrices designed for MU-MISO systems to maximize the sum rate. Unlike traditional algorithms, such as MO-AltMin, SDR-AltMin, and PE-AltMin—which suffer from high computational complexity—and OMP, which achieves lower sum rate performance, all conventional algorithms exhibit degradation and limited adaptability to imperfect CSI, making them impractical for real-time implementation. In contrast, the proposed DL-HBF method achieves an acceptable sum rate with low latency and its superior robustness to channel estimation errors, which makes it suitable for practical deployment in real-time wireless systems. We validate these methods in a realistic channel model to ensure robustness and applicability. On the other hand, we make procedures to generate the dataset in a short time and reduce the training time compared to existing DL-based hybrid beamforming methods. Our results underscore the potential for DL in hybrid beamforming to provide next-generation wireless systems with high sum rates, low execution time and its strong capability in mapping imperfect CSI to the near-optimal hybrid beamforming matrices, where its application demands that. Future work may discuss extending this system to a complex system configuration, such as multi-cell and massive MIMO scenarios.

## Data Availability

All data generated or analysed during this study are included in this published article
